# Limited dCTP Availability Accounts for Mitochondrial DNA Depletion in Mitochondrial Neurogastrointestinal Encephalomyopathy (MNGIE)

**DOI:** 10.1371/journal.pgen.1002035

**Published:** 2011-03-31

**Authors:** Emiliano González-Vioque, Javier Torres-Torronteras, Antoni L. Andreu, Ramon Martí

**Affiliations:** 1 Laboratori de Patologia Mitocondrial, Institut de Recerca Hospital Universitari Vall d'Hebron, Universitat Autònoma de Barcelona, Barcelona, Spain; 2 Biomedical Network Research Centre on Rare Diseases (CIBERER), Instituto de Salud Carlos III, Madrid, Spain; Max Planck Institute for Biology of Aging, Germany

## Abstract

Mitochondrial neurogastrointestinal encephalomyopathy (MNGIE) is a severe human disease caused by mutations in *TYMP*, the gene encoding thymidine phosphorylase (TP). It belongs to a broader group of disorders characterized by a pronounced reduction in mitochondrial DNA (mtDNA) copy number in one or more tissues. In most cases, these disorders are caused by mutations in genes involved in deoxyribonucleoside triphosphate (dNTP) metabolism. It is generally accepted that imbalances in mitochondrial dNTP pools resulting from these mutations interfere with mtDNA replication. Nonetheless, the precise mechanistic details of this effect, in particular, how an excess of a given dNTP (e.g., imbalanced dTTP excess observed in TP deficiency) might lead to mtDNA depletion, remain largely unclear. Using an *in organello* replication experimental model with isolated murine liver mitochondria, we observed that overloads of dATP, dGTP, or dCTP did not reduce the mtDNA replication rate. In contrast, an excess of dTTP decreased mtDNA synthesis, but this effect was due to secondary dCTP depletion rather than to the dTTP excess in itself. This was confirmed in human cultured cells, demonstrating that our conclusions do not depend on the experimental model. Our results demonstrate that the mtDNA replication rate is unaffected by an excess of any of the 4 separate dNTPs and is limited by the availability of the dNTP present at the lowest concentration. Therefore, the availability of dNTP is the key factor that leads to mtDNA depletion rather than dNTP imbalances. These results provide the first test of the mechanism that accounts for mtDNA depletion in MNGIE and provide evidence that limited dNTP availability is the common cause of mtDNA depletion due to impaired anabolic or catabolic dNTP pathways. Thus, therapy approaches focusing on restoring the deficient substrates should be explored.

## Author Summary

Mitochondria are subcellular organelles that constitute the main energy supply within the cell. They contain their own DNA, which should be continuously replicated to ensure the correct mitochondrial function. Several mitochondrial diseases are caused by genetic defects that compromise this replication and result in mitochondrial DNA depletion. In most cases, these genetic defects block the synthesis of dATP, dGTP, dCTP, and dTTP, the 4 nucleotides needed for mitochondrial DNA replication. However, for one of these disorders (mitochondrial neurogastrointestinal encephalomyopathy, MNGIE), the biochemical pathways needed to synthesize them are intact, but degradation of dTTP is genetically blocked, leading to dTTP accumulation. We investigated the biochemical mechanisms through which the dTTP excess leads to mitochondrial DNA depletion in MNGIE, and we found that the delay of mitochondrial DNA replication rate observed when dTTP is in excess is not caused by this excess in itself. Instead, the dTTP overload produces a secondary dCTP depletion that actually delays mitochondrial DNA replication. Therefore, the common factor accounting for mitochondrial DNA depletion in these disorders is the limited availability of one or more nucleotides. This indicates that strategies to provide nucleotides to patients' mitochondria should be explored as a possible treatment for these fatal disorders.

## Introduction

Mitochondrial DNA depletion syndrome refers to a group of inherited metabolic disorders that considerably differ in their clinical expression and genetic origin [1]. These are rare, but severe diseases with no effective treatment, and patients die in the early infancy in most cases. Four of the 9 genes known to be associated with this group of disorders to date are directly involved in the metabolism of deoxyribonucleoside triphosphates (dNTPs), required for mtDNA replication [2]–[5]. Two of these genes (*TK2*, Entrez Gene ID 7084, and *DGUOK*, Entrez Gene ID 1716) encode the mitochondrial enzymes thymidine kinase 2 (TK2) and deoxyguanosine kinase (dGK), which catalyze the first step in mitochondrial salvage of pyrimidine and purine deoxyribonucleosides, respectively. *RRM2B* (Entrez Gene ID 50484) encodes the p53-inducible small subunit (p53R2) of ribonucleotide reductase (RNR), the key enzyme in *de novo* synthesis of deoxyribonucleotides. A p53-independent small subunit of RNR is highly expressed in dividing cells, but is degraded and its expression downregulated in quiescent cells; thus, the *de novo* supply of dNTPs for mitochondrial DNA (mtDNA) replication is dependent on p53R2 [6]. Mutations in the fourth gene, *TYMP* (Entrez Gene ID 1890), encoding thymidine phosphorylase, cause mitochondrial neurogastrointestinal encephalomyopathy (MNGIE, OMIM ID #603041) [4]. Dysfunction of this cytosolic enzyme causes expansion of the dTTP pool [7].

**Figure 1:**
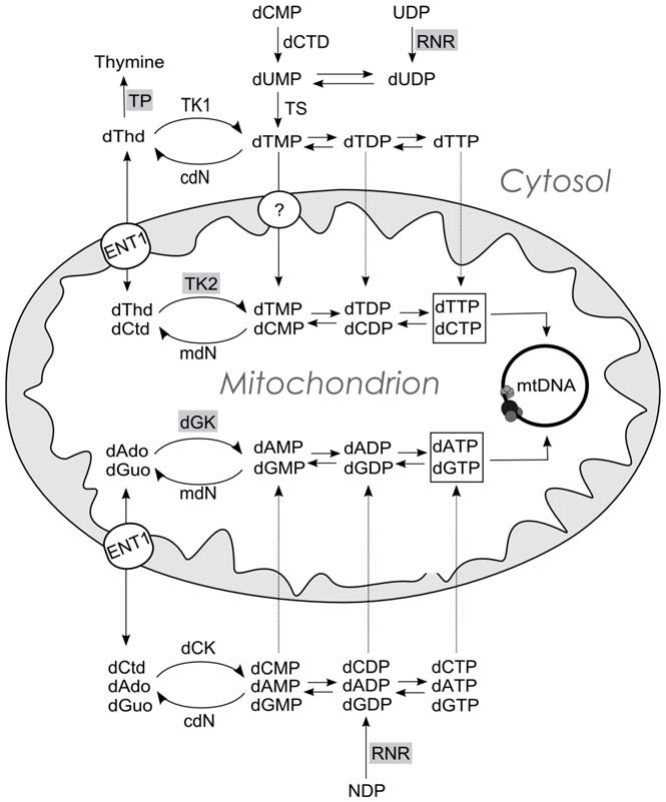
Schematic representation of the metabolic pathways that supply dNTPs for mtDNA replication. dThd: thymidine; dCtd: deoxycytidine; dAdo: deoxyadenosisne; dGuo: deoxyguanosine; TP: thymidine phosphorylase; TK1 (cytosolic) and TK2 (mitochondrial) thymidine kinases; dGK: deoxyguanosine kinase; dCK: deoxycytidine kinase; cdN and mdN: cytosolic and mitochondrial deoxynucleotidases; dCTD: dCMP deaminase; TS thymidylate synthase; RNR: ribonucleotide reductase. ENT1: equilibrative nucleoside transporter 1, which has been found in human mitochondria [30], [31]. The circle with a question mark indicates evidence of a highly concentrative dTMP transport [32] by an unidentified carrier. A dCTP transport activity has also been reported [21]. Enzymes involved in mtDNA depletion syndrome are highlighted in grey.

dNTP supply for mtDNA replication (Figure 1) depends on intramitochondrial salvage of deoxyribonucleosides, or on the mitochondrial import of deoxyribonuceloside phosphates from the cytosol, made *de novo* from ribonucleotide reduction or from cytosolic deoxyribonucleoside salvage. These pathways are interconnected and the relative contributions of each pathway depend on a number of different factors, most importantly, the cell cycle (reviewed in [8]). It is generally assumed that imbalances in the mitochondrial dNTP pools interfere with the correct mtDNA replication and/or repair [1], [8], but there is no experimental evidence on the particular consequences of such imbalances on mtDNA replication. The precise mechanistic details of this effect remain largely unknown; the foreseeable depletion of dNTPs resulting from a loss of function of TK2, dGK and RNR fits well with mtDNA depletion syndrome, since dNTPs are the substrates of mtDNA replication, but it is unclear how an excess of a given dNTP, specifically the dTTP expansion associated to thymidine phosphorylase dysfunction in MNGIE, might lead to mtDNA depletion. Other groups have reported the effects of thymidine addition on dNTP pools in cell culture. It has long been known that, in cycling cells, millimolar concentrations of thymidine cause pronounced dCTP depletion due to the allosteric effects of an expanded dTTP pool on RNR, which strongly inhibit the nuclear DNA replication rate [9]. More recently, several studies have shown that micromolar thymidine concentrations, similar to those found in MNGIE patients, result in slight to moderate cytosolic and mitochondrial dCTP depletion in cycling and quiescent cultured cells [10]–[12].

Here, we studied the consequences of dNTP imbalances on mtDNA replication in two different experimental models, and found that the limited availability of one dNTP is a common mechanism accounting for mtDNA depletion even under dTTP excess. The replication is limited by the availability of the dNTP present at the lowest concentration and, in the case of dTTP excess, secondary dCTP depletion accounts for the reduction of mtDNA replication rate.

## Results/Discussion

We used an *in organello* replication approach [13] to investigate whether excess of any of the 4 dNTPs affects mtDNA replication, measured by incorporation of radiolabel from [^3^H]-dNTP or [α-^32^P]-dNTP into mtDNA. Through controlled addition of the 4 dNTPs to the reaction, this model enabled us to study the consequences of experimentally designed mitochondrial dNTP imbalances on mtDNA replication. To assess modifications of the intra-mitochondrial dNTP pool resulting from addition of each dNTP to a mitochondrial suspension, mitochondria were extracted from liver of C57BL/6J mice, endogenous dNTPs were measured, and the effect of adding no dNTPs, 1 µM of all 4 dNTPs, and 100 µM of each dNTP was studied (Table 1). All 4 endogenous dNTPs were partially depleted over 2 hours of *in organello* reaction. While dCTP was the most abundant endogenous nucleotide in fresh mitochondria, it became the least plentiful at the end. Addition of 1 µM of extramitochondrial dNTPs led to 4-fold to 7-fold increases in intramitochondrial dNTPs after 2-hour incubation. Addition of 100 µM of dTTP, dCTP, or dGTP resulted in a 100-fold to 270-fold increase in the amount of each nucleotide; when 100 µM dATP was added, a much larger increase (∼600-fold) was observed in the intramitochondrial amount of this nucleotide. These results were independently investigated by measuring the radioactivity incorporated into mitochondria when 100 µM of tritium-labeled dNTPs were added to the reaction. We found that 2 to ∼6.5 times more dATP (or its dephosphorylated derivatives) entered the mitochondria than dTTP, dCTP, or dGTP (Figure S1). The enhanced transport observed when dATP was added in large excess might be mediated by the abundant ADP/ATP carrier; it has long been known that dADP and dATP can be substrates of this ribonucleotide transporter [14], [15]. Interestingly, comparison of these results with those of Table 1 shows that most dNTP molecules transported into mitochondria are partially or totally dephosphorylated.

**Table 1:** Effect of exogenous dNTP addition on intramitochondrial dNTP pools.

	Before reaction	After 2 h reaction, when added:						
				1 µM each dNTP plus 100 µM of:				
	No addition	No addition	1 µM each dNTP	dATP	dTTP	dCTP	dGTP	dThd
**dATP**	1.41±0.35	0.63±0.14	4.62±0.22	405±48	4.66±0.91	ND	ND	3.55±0.73
**dTTP**	2.28±0.33	0.30±0.06	1.31±0.60	ND	37.5±4.5	ND	ND	5.25±1.71 ^**^
**dCTP**	7.53±1.57	0.22±0.09	1.37±0.58	ND	0.85±0.20 ^*^	59.5±11.1	ND	0.68±0.37 ^**^
**dGTP**	1.22±0.19	0.45±0.07	2.51±0.81	ND	2.30±0.70	ND	52.8±5.4	2.02±0.44

Results expressed in pmoles/mg prot, as mean±SD of N = 3 experiments, except for (*) (N = 5) and (**) (N = 4). ND: not determined.

**Figure 2:**
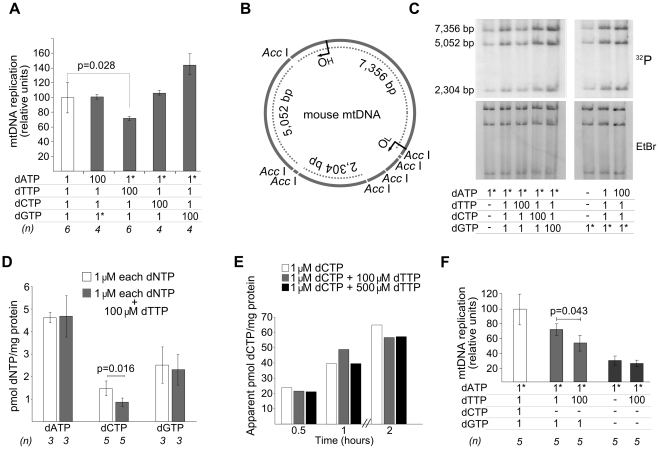
dTTP excess causes a decrease of *in organello* mtDNA synthesis associated with secondary dCTP depletion. Concentrations (µM) of dNTPs added to the reaction are indicated in the tables attached to the panels. Dashes: no dNTP addition. Asterisks: radiolabeled nucleotide. All results (except panel E) were obtained after 2 hours of *in organello* reaction. Bars represent mean±SD. A. Effect of an excess of each dNTP on mtDNA replication. Tritium-labeled dATP or dGTP were used. The reference result (open bar) is plotted as the mean of all the experiments, equaled to 100%, the error bar indicates the SD as percentage. *P* values obtained with the Wilcoxon T-test. B. Representation of *Acc*I-digested mouse mtDNA. O_H_, origin of heavy strand replication. O_L_, origin of light strand replication. Dotted lines, fragments visualized in the gels in panel C, which show the bands resulting from the *Acc*I-digestion; [α^32^P]-labeled DNA was extracted, *Acc*I-digested, and electrophoresed. The top (^32^P) and bottom (EtBr) gels show the radioactive signal and ethidium bromide staining, respectively. The even distribution of radioactivity in the 3 bands (here and Figure 3B) indicates that *de novo* replication in our model is distributed thorough the entire mtDNA, in contrast to other situations in which the 7S-fragment is preferentially replicated [33]. D. Effect of dTTP excess on mitochondrial dNTP pools. *P* values obtained with Mann-Whitney U test. E. Effect of dTTP excess on transport of radioactive label from exogenous 1 µM [5,5′-^3^H]dCTP to mitochondria. Radioactivity of the mitochondrial pellet was measured and apparent pmoles were estimated from the specific radioactivity of dCTP. F. dTTP-induced decrease of mtDNA replication in the absence of exogenous dCTP and dGTP. *P* values obtained with Wilcoxon T-test.

Using this model, a 100-fold increase of dATP or dCTP concentrations in the reaction did not produce detectable changes in mtDNA replication, measured by incorporation of ^3^H or ^32^P into mtDNA, whereas excess dGTP induced a 45% increase (Figure 2A, 2B, 2C). The same excess of dTTP, however, caused a significant 25% decrease in mtDNA replication. Thus, only dTTP excess had a negative effect on mtDNA replication. In an early study it was reported that addition of thymidine to Chinese hamster ovary cells leads to an increase in the dTTP pool and cessation of nuclear DNA replication, resulting from dCTP depletion [9]. More recently, several reports have shown reductions in cellular or mitochondrial dCTP in parallel to dTTP expansion following thymidine overload in cultured cells [10]–[12]. We then measured the 4 dNTPs in the presence of dTTP overload to determine whether additional mitochondrial dNTP imbalances were produced in our model. A significant decrease in dCTP concentration was observed in association with dTTP excess, with no effect on dATP or dGTP (Table 1 and Figure 2D). The dCTP contraction was not caused by an inhibitory effect of the large dTTP excess on transport of the exogenously provided dCTP because incorporation of [5,5′-^3^H]dCTP label into mitochondria was not affected by increasing amounts of dTTP (Figure 2E).

We tested the influence of dTTP overload on mtDNA replication in 2 additional conditions: 1) without adding dCTP and 2) without adding either dCTP or dGTP (Figure 2F). The inhibitory effect of dTTP on mtDNA replication was also observed in the absence of exogenous dCTP, thus confirming that this effect is not caused by inhibition of dCTP transport by an excess of dTTP. The effect of dTTP overload (100 µM added) on the endogenous dCTP pool at the end of the reaction was tested in 5 independent experiments, which also revealed dCTP contraction (by 56.4%±20.6%; P = 0.016, Mann-Whitney U test). In contrast, dTTP overload did not influence mtDNA replication when exogenous dCTP and dGTP were both omitted, probably because endogenous dGTP is the limiting substrate of the reaction. This is consistent with the fact that dGTP is present in the lowest concentration in freshly isolated mitochondria (Table 1). In keeping with this concept, dGTP was the only dNTP that, in excess, stimulated mtDNA replication (Figure 2A).

**Figure 3:**
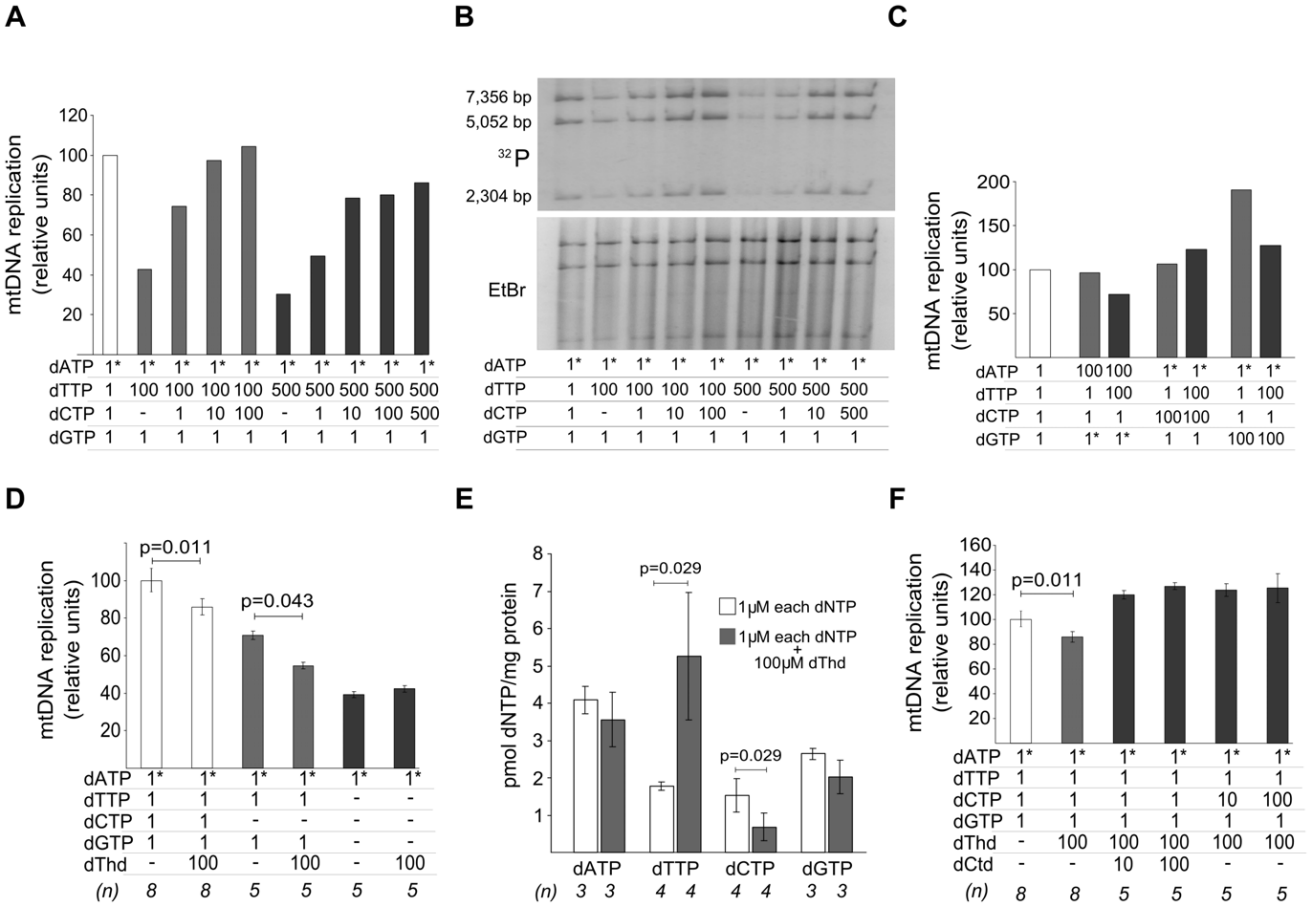
dTTP- and thymidine-induced decrease in mtDNA replication is prevented by dCTP supplementation. Concentrations (µM) of dNTP or nucleosides added to the reaction are indicated in the tables attached to the panels. Dashes: no addition. Asterisks: radiolabeled nucleotide. All results were obtained after 2 hours of *in organello* reaction. Bars represent mean±SD. dThd: thymidine; dCtd: deoxycytidine. A, B. mtDNA synthesis in the presence of increasing amounts of dTTP and dCTP; ^3^H-labeled DNA was quantified in (A) or ^32^P-labeled DNA was *Acc*I-digested and resolved in (B). C. Supplementation with dCTP excess (but not dATP or dGTP excess) prevented dTTP-induced decrease in mtDNA replication. D. Effect of thymidine excess on mtDNA replication. *P* values obtained with Wilcoxon T-test. E. Effect of thymidine excess on mitochondrial dNTP pools. *P* values obtained with Mann-Whitney U test. F. Thymidine-induced decrease of mtDNA replication was prevented by supplementation with excess of deoxycytidine or dCTP. *P* values obtained with Wilcoxon T-test.

Two alternative mechanisms could account for the negative effect of dTTP excess on mtDNA replication: 1) dTTP overload, in itself, may slow down the replication machinery, or 2) secondary dCTP depletion may restrict replication because dCTP becomes the limiting substrate. To test these alternatives, we attempted to restore mtDNA replication by supplying additional dCTP to a reaction under dTTP excess. As shown in Figure 3A, 3B, the dose-dependent negative effect of dTTP on mtDNA replication was prevented, also in a dose-dependent manner, by dCTP supplementation. Addition of dATP or dGTP failed to prevent the negative effect of dTTP (Figure 3C), even though dGTP had an independent positive effect, as was mentioned above (Figure 2A). This positive effect was not due to a dGTP-induced dCTP increase, because the addition of 100 µM dTTP induced similar reductions of dCTP levels both in the absence (0.85±0.20 pmol/mg prot, from Table 1) and in the presence (0.71±0.20 pmol/mg prot; N = 3) of 100 µM dGTP. Monitoring mtDNA replication over 2 hours showed that the dGTP-induced positive effect was very pronounced in the initial phase (first 30 min), and very reduced or negligible during the second hour (Figure S2), suggesting that, in our model, dGTP concentration is the limiting factor only in the initial phase of the replication.

These results demonstrate that the decrease in mtDNA replication occurring under dTTP overload in our model is caused by secondary dCTP depletion, rather than by dTTP excess. We postulate that progression of mtDNA replication *in organello* is limited by the availability of each dNTP substrate. This notion is supported by 3 observations: 1) dGTP, the endogenous nucleotide in lowest concentration, was the only dNTP that, in excess, stimulated mtDNA replication (Figure 2A), 2) no positive or negative effect was observed by dTTP excess in the absence of exogenous dGTP (Figure 2F), and 3) mtDNA replication was sensitive to removal of each separate exogenous dNTP (Figure S3).

The primary biochemical imbalance in thymidine phosphorylase-deficient MNGIE patients is systemic accumulation of thymidine, which leads to expansion of the dTTP pool in cultured cells [10]–[12] and *in vivo* [7]. The results reported above indicate that most triphosphate molecules entering the mitochondria lose one or more phosphates. Therefore, we tested whether the effect of thymidine overload in our *in organello* reaction caused effects similar to those observed with dTTP excess (Figure 3D, 3E, 3F). Thymidine overload slowed down mtDNA replication, with and without addition of 1 µM dCTP (Figure 3D) and, again, this effect disappeared when dGTP addition was omitted. Addition of extramitochondrial thymidine increased intramitochondrial dTTP, revealing active *in organello* phosphorylation through TK2, and decreased dCTP, paralleling the results obtained with dTTP excess (Figure 3E). dATP and dGTP also showed a slight trend to be reduced, but far from the ∼50% reduction observed for dCTP. Noteworthily, while intramitochondrial dTTP expansion was moderate (3-fold) and far from the nearly 30-fold increase observed when 100 µM of dTTP was added (Table 1), the extent of the dCTP decrease was very similar, suggesting that dCTP contraction may be caused mainly by thymidine rather than by dTTP. Addition of deoxycytidine or dCTP restored mtDNA replication in the presence of thymidine overload (Figure 3F), thus confirming that thymidine excess, in itself, does not slow down mtDNA replication.

**Figure 4:**
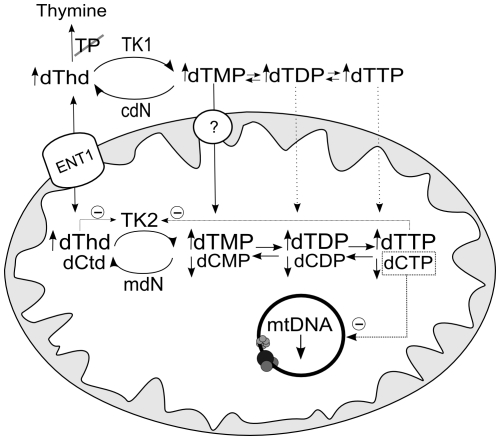
Hypothetical mechanism accounting for decrease in mtDNA replication caused by thymidine overload. TK2-catalyzed deoxycytidine phosphorylation is inhibited competitively by thymidine and noncompetitively by dTTP [16], leading to depletion of dCTP, which becomes the limiting dNTP for mtDNA replication. ENT1: equilibrative nucleoside transporter 1, which has been found in human mitochondria [30], [31]. The circle with a question mark indicates evidence of a highly concentrative dTMP transport [32] by an unidentified carrier. mdN and cdN: mitochondrial and cytosolic deoxynucleotidases. dThd: thymidine; dCtd: deoxycytidine.

Phosphorylation of thymidine and deoxycytidine is catalyzed by the same mitochondrial kinase, TK2. Studies in the human enzyme have shown that TK2-catalyzed deoxycytidine phosphorylation is competitively inhibited by thymidine, and thymidine phosphorylation is also inhibited (less effectively) by deoxycytidine, while both are feedback-inhibited by dTTP and dCTP [16] (Figure 4). Therefore, we would expect that dCTP excess and the deoxycytidine generated by dCTP dephosphorylation, would also reduce mtDNA replication. In our model, dCTP excess did not affect mtDNA replication in the presence of dTTP (Figure 2A and Figure 3C). However, when we repeated the experiment in the absence of exogenous dTTP (Figure S4), addition of 100 µM dCTP decreased mtDNA replication by 44.4% (±4.3%), supporting the idea that the effects produced by dTTP and dCTP excesses are both TK2-mediated.

Secondary dCTP depletion due to TK2 inhibition has been suggested to occur in MNGIE [10], [17]. The present report provides the first direct evidence that thymidine-induced mitochondrial dCTP contraction, previously observed by others [10]–[12], delays mtDNA replication in vitro, rather than dTTP excess. We found that ∼40% contraction of the mitochondrial dCTP pool leads to decreased mtDNA replication *in organello*, while dTTP increases as high 30-fold do not produce observable effects. Previous *in vivo* observations are consistent with this mechanism. Brain and liver mitochondrial dTTP were both found to be increased in the double *Tymp/Upp1* knockout murine model of MNGIE, but mitochondrial dCTP depletion was only seen in brain. Interestingly, brain, but not liver, had mtDNA depletion [7], supporting the notion that dCTP depletion, and not dTTP excess, is associated with mtDNA copy number decreases.

**Figure 5:**
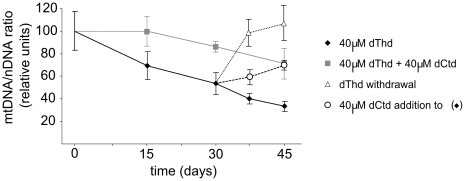
Thymidine-induced mtDNA depletion is prevented by deoxycytidine supplementation in human cultured cells. Influence of thymidine and deoxycytidine supplementation on mtDNA copy number in quiescent primary human skin fibroblasts in culture. Cells were collected at the times indicated and mtDNA/nDNA ratio was assessed. Results are plotted as percentages of the ratios obtained for parallel cultures with no nucleoside addition. Error bars represent standard error of the mean of N independent experiments (N = 4 for squares and rhombs; N = 3 for circles and triangles). dThd: thymidine; dCtd: deoxycytidine.

Thymidine overload is reported to cause mtDNA depletion in human cultured cells [11]. Using the same model, we tested whether addition of deoxycytidine prevents the negative effects of thymidine in human cells. We found that mtDNA depletion caused by 40 µM thymidine is prevented by co-treatment with 40 µM deoxycytidine (Figure 5). Similarly, after 30 days under thymidine overload, mtDNA-depleted cells gradually recovered mtDNA copy number when deoxycytidine was added to the medium. Therefore, our results demonstrate that the negative effect of thymidine on mtDNA in cultured human cells can be prevented by deoxycytidine.

Despite the increasing number of studies investigating the molecular mechanisms involved in mtDNA depletion syndrome, many unsolved questions require further effort [1], [8], [18]. In the case of MNGIE, other groups have developed models to study the effect of thymidine overload on dNTP pools and on mtDNA replication in cell culture [10]–[12]. Interestingly, all these studies revealed moderate cytosolic and mitochondrial dCTP depletion secondary to thymidine overload, as well as diverse effects on mtDNA. After a long treatment (8 months) of HeLa cells with thymidine, Song et al [12] found multiple deletions in mtDNA, but failed to detect depletion, probably because the study was performed using a highly proliferating cell line. More recently, Pontarin et al [11] tested the effects of thymidine on quiescent lung and skin fibroblasts, and mtDNA depletion, but not deletions, was observed. Here, we have tried to address how dTTP expansion and dCTP contraction contribute to the reduction of mtDNA copy number using isolated murine mitochondria. This model allowed us to study in controlled conditions the changes of the replication rates induced by designed dNTP imbalances. Our results indicate that mitochondrial dNTP availability is the key factor for mtDNA maintenance in mtDNA depletion syndrome caused by mutations in *TYMP*, *TK2*, *DGUOK*, and *RRM2B*. These last 3 genes directly compromise the *salvage* or *de novo* supply of mitochondrial dNTPs, in agreement with the clinical severity of cases with *TK2*, *DGUOK*, and *RRM2B* mutations. In MNGIE patients, however, all anabolic steps that provide mitochondrial dNTPs for mtDNA replication are fully functional, and the main factor accounting for the impaired replication is believed to be dTTP excess caused by thymidine phosphorylase deficiency. While this is likely true for the multiple deletions or somatic point mutations generated by a dTTP-driven next nucleotide effect [19], [20], the experimental evidence presented here indicates that mitochondrial dCTP depletion secondary to thymidine excess accounts for mtDNA depletion, rather than dTTP excess.

Although using isolated mitochondria allowed us to easily study the effect of dNTP imbalances on mtDNA replication, some limitations derived from this simplified system should prompt one to be cautious when interpreting the results. For example, isolated mitochondria lack deoxycytidine kinase (dCK), the cytosolic enzyme that catalyzes the phosphorylation of all deoxyribonucleosides except deoxyuridine and thymidine, which do not inhibit deoxycytidine phosphorylation by dCK. Our in organello system is also lacking *de novo* dCDP (and other dNDPs) synthesis via RNR. In cultured cells and in vivo, these pathways could compensate or reduce the effect of the mitochondrial dCTP decay studied here, because deoxyribonucleotides can be imported from the cytosol to mitochondria [21]. However, as mentioned above, mitochondrial dCTP depletion has been reported in cell culture and in *in vivo* models of MNGIE [7], [10]–[12], indicating that, at least in some cells and tissues, this compensation does not suffice to normalize mitochondrial dCTP levels. Further support came from our recovery experiments in quiescent fibroblasts: cotreatment with deoxycytidine largely prevented thymidine-induced mtDNA, indicating that dCTP contraction should be the cause of the thymidine-induced mtDNA depletion not only in isolated mitochondria but also in cultured cells.

Therefore, the common biochemical condition in mitochondrial DNA depletion syndrome due to imbalanced dNTP pools (including MNGIE) is a limited availability of one or more substrates for mtDNA replication. Based on this idea, therapy approaches for this group of disorders should focus on supplying the specific substrates that are depleted in each enzyme defect. Previous studies have experimentally examined the potential benefits of biochemically by-passing these enzyme defects. Supplementation with purine deoxyribonucleoside monophosphates prevented mtDNA depletion in dGK-deficient cultured cells [22], [23]. In the case of MNGIE, the treatment relies on providing thymidine phosphorylase activity through allogeneic hematopoietic stem cell transplantation [24], but preventing mitochondrial dCTP depletion could be a complementary approach. Deoxycytidine supply would compensate for thymidine competition for TK2-mediated phosphorylation, or the nucleoside would be phosphorylated by dCK resulting in more cytosolic deoxycytidine nucleotides available to be imported by mitochondria. Both mechanisms would increase mitochondrial dCTP levels, thus preventing the negative effects of dCTP depletion on mtDNA replication. However, the optimal way to provide this precursor is not obvious, because of the complicated interconnections between the enzymes of dNTP metabolism. Enzymatic deamination would limit the availability of deoxycytidine, as happens with deoxycytidine analogues [25]. Cytidine deaminase inhibitors, alone or in combination with deoxycytidine, would be an alternative [25], because they would limit the conversion of endogenous or supplied deoxycytidine to deoxyuridine. The availability of a murine model of the disease [7] will enable us to test the potential benefits of these approaches, and to find the way to avoid possible pitfalls.

## Materials and Methods

This study was carried out in accordance with the rules established by the *Generalitat de Catalunya* for the Care and Use of Laboratory Animals. The protocol was approved by the Committee on the Ethics of Animal Experiments of the *Institut de Recerca Hospital Universitari Vall d'Hebron* (Permit Number: 58/09).

### *In organello* replication

Mitochondria were isolated as described [26] with minor modifications. Two to 3-month-old male C57BL/6J mice were killed by cervical dislocation and the liver was rapidly removed and chilled in homogenization buffer (4 mL/g tissue, 320 mM sucrose, 1 mM EDTA, 10 mM Tris-HCI, pH 7.4). All further operations were carried out at 2–4°C using sterile solutions. The liver was cut in small pieces, homogenized in a Dounce homogenizer (4 up-and-down strokes), and spun at 1,000×g for 5 min. The supernatant was aliquoted in Eppendorf tubes and centrifuged at 13,000×g for 2 min. The mitochondrial pellets were washed 3 times in homogenization buffer and once in incubation buffer (25 mM sucrose, 75 mM sorbitol, 100 mM KCl, 10 mM K_2_HPO_4_, 0.05 mM EDTA, 5 mM MgCl_2_, and 10 mM Tris-HCl, pH 7.4). Lastly, the mitochondrial pellet was resuspended in 1 mL of incubation buffer and protein concentration was determined (Coomassie Plus Assay Kit, Thermo Scientific, Rockford IL). Samples containing 500 µg of protein were resuspended in 250 µL of incubation buffer supplemented with 1 mM ADP, 10 mM glutamate, 2.5 mM malate and 1 mg/mL fatty acid-free bovine serum albumin, as well as dNTPs at the concentrations indicated for each experiment. The radiolabeled nucleotides used were [8-^3^H]dATP, [8-^3^H]dGTP, or [5,5′-^3^H]dCTP (between 12–21 Ci/mmol) and [α-^32^P]dATP or [α-^32^P]dGTP (100 Ci/mmol), depending on the experiment. In these conditions, mitochondria were incubated at 37°C in a rotary shaker (*in organello* reaction) for 2 h, unless otherwise indicated. Mitochondria were then pelleted (13,000×g for 1 min) and washed twice with 10% glycerol, 0.15 mM MgCl_2_, and 10 mM Tris-HCl (pH 6.8). DNA was extracted from this pellet as described [27]. Briefly, the pellet was lysed with 500 µL of 20 mM Hepes-NaOH, (pH 7.4) 75 mM NaCl, 50 mM EDTA, 20 µg/mL proteinase K, and incubated at 4°C for 45 min. Then, 17 µL of 30% lauryl maltoside was added and the mixture was incubated at 50°C for 45 min. Twenty-five µL of the homogenate was used for scintillation counting (Beckman Coulter LS 6500, Brea, CA) to quantify the total label within mitochondria. These data allowed us to rule out that differences in DNA labeling were due to differential amounts of radioactive nucleotide transported into mitochondria. The remaining mitochondrial homogenate was used for phenol-chloroform extraction, and DNA was dissolved in TE buffer (1 mM EDTA, 10 mM Tris-HCl; pH 7.5) and quantified (Quant-iT PicoGreen dsDNA Reagent, Invitrogen). The DNA-incorporated radiolabel was measured by scintillation counting. De novo mtDNA replication was quantified as the apparent fmoles of ^3^H-labeled nucleotide per ng DNA, calculated from the specific radioactivity of the radiolabeled nucleotide used.

In each assay, a reaction with 1 µM of each of the 4 dNTPs was processed. This reference point was considered 100% and the remaining results were expressed as a percentage. When ^32^P-labeled dATP or dGTP were used, DNA was dissolved in 20 mM Tris-acetate, 50 mM potassium acetate, 10 mM magnesium acetate, and 1 mM dithiothreitol, pH 7.9, and digested with *Acc*I (New England Biolabs, Ipswich, MA). The product was resolved in 1% agarose gel in 40 mM Tris-acetate and 1 mM EDTA, pH 8.0, stained with ethidium bromide to capture the total DNA image, then washed in TE buffer and dried under vacuum. The radiolabeled bands, representing *de novo* synthesized DNA, were visualized by autoradiography and quantified (Image J software, NIH).

### Mitochondrial dNTP content determination

Mitochondria (500 µg of protein) were treated with 200 µL of 60% methanol and kept at −20°C for 2 h. The protein was pelleted at 25,000×g for 10 min and the supernatant incubated at 100°C for 3 min, dried under speed vacuum and redissolved in 20–100 µL of water, depending on the expected dNTP concentration (dNTP extract). dNTP content was determined using a polymerase-based method [28] with minor modifications. Briefly, 20 µL of reaction mixture contained 5 µL of dNTP extract in 40 mM Tris-HCl, pH 7.4, 10 mM MgCl_2_, 5 mM dithiothreitol, 0.25 µM oligoprimer, 0.75 µM [8-^3^H]dATP, 12–21 Ci/mmol (or [methyl-^3^H]dTTP for the dATP assay) and 1.7 units of Thermo Sequenase DNA Polymerase (GE Healthcare). Reaction mixtures with aqueous dNTP standards were processed in parallel. After incubation at 48°C for 60 min, 18 µL of the mix was spotted on a Whatman DE81 paper and left to dry. The filters were washed 3 times for 10 min with 5% Na_2_HPO_4_, once with water, once with absolute ethanol, and left to dry again. The retained radioactivity was determined by scintillation counting, and dNTP amounts calculated from interpolation on the calibration curves. To ensure the reliability of the results, triplicates of 2 different dilutions of each dNTP extract (usually undiluted and 1∶3 water-diluted) were processed in each independent experiment. When dATP was determined in extracts with large amounts of dTTP (37.5±4.5 pmol/mg protein, Table 1), which significantly dilutes the label ([methyl-^3^H]dTTP) used for the assay [28], the dilution was taken into account and the appropriate correction factor was applied to the results.

### Cell culture

Human primary skin fibroblasts (passage 4 or lower) from 4 healthy controls were seeded in 3.5-cm diameter plates (100,000 cells per plate) and expanded in Dulbecco's modified Eagle's medium with 4.5 g/L glucose, supplemented with 2 mM L-glutamine, 100 U/mL penicillin and streptomycin, and 10% dialyzed FBS (Invitrogen) in a humidified incubator at 37°C and 5% CO_2_. Four days after tight confluence was reached, FBS was reduced to 0.1%. Four days later (day 0) different plates were maintained in the following conditions: 1) no added nucleosides, 2) 40 µM thymidine, and 3) 40 µM thymidine+40 µM deoxycytidine. At day 30, 2 additional conditions were generated: 4) thymidine withdrawal from condition 2, and 5) 40 µM deoxycytidine added to condition 2. Media was replaced every 3 days to ensure that thymidine and deoxycytidine concentrations were always between 10 and 40 µM, as tested by HPLC. On different days, cells were harvested by trypsinization, washed with phosphate-buffered saline, pelleted and stored at −20°C until DNA isolation.

### Quantification of mtDNA copy number

DNA was isolated from cell pellets (QIAampDNA Mini kit, Qiagen), dissolved in TE buffer, and quantified (NanoDrop Spectrophometer, Thermo Scientific). Relative quantitation of mtDNA (12S rRNA gene) versus nuclear DNA (*RNase P* single copy gene) was performed using an ABI PRISM® 7500 real-time PCR system (Applied Biosystems). Custom designed primers and probe for the 12S rRNA gene were used for assessing mtDNA copy number, which was expressed as the ratio over the copy number of the *RNase P* single copy nuclear gene, as previously described [29].

## Supporting Information

Figure S1 Transport of radioactive label from exogenous 100 µM [8-^3^H]dATP, [8-^3^H]dGTP, [5,5′-^3^H]dCTP or [methyl-^3^H]dTTP into mitochondria after 2 hours of in organello reaction. Radioactivity of the mitochondrial pellet was measured and apparent pmoles were estimated from the specific radioactivity of the dNTPs. Concentrations (µM) of dNTPs added to the reaction are indicated in the attached table. Asterisks: radiolabeled nucleotide. Bars represent mean±SD.
(PDF)

Figure S2 Monitoring of mtDNA synthesis over 2 hours with dGTP and dTTP excess. Concentrations (µM) of dNTPs added to the reaction are indicated in the table on the right. The replication observed after 2 hours of reaction with 1 µM each dNTP added (rhombs) is considered the reference point (100%). Error bars represent ±SD (N = 3). Replication rates in the table at the bottom are expressed as the percentage of replication per minute.
(PDF)

Figure S3 Effect of removing each single exogenous dNTP on mtDNA synthesis. Concentrations (µM) of dNTPs added to the reaction are indicated in the attached table. Dashes: no dNTP addition. Asterisks: radiolabeled nucleotide. Results were obtained after 2 hours of in organello reaction.
(PDF)

Figure S4 Effect of dCTP excess on mtDNA synthesis when exogenous dTTP (or dTTP and dGTP) was omitted in the in organello reaction. The dCTP-induced decrease of mtDNA synthesis disappeared when dGTP was omitted, likely because dGTP became the limiting substrate, as is suggested in Figure 2A and 2F, and Figures S2 and S3. Bars represent mean±SD. The reference result (open bar) is plotted as the mean of all the experiments, equaled to 100% and the error bar indicates the SD as percentage. P values obtained with the Wilcoxon T-test.
(PDF)
