# Supplementary figures and images for: Limited dCTP Availability Accounts for Mitochondrial DNA Depletion in Mitochondrial Neurogastrointestinal Encephalomyopathy (MNGIE)
Source: PLoS Genet. 2011 Mar 31;7(3):e1002035. doi: 10.1371/journal.pgen.1002035 (PMC3069123; doi:10.1371/journal.pgen.1002035)

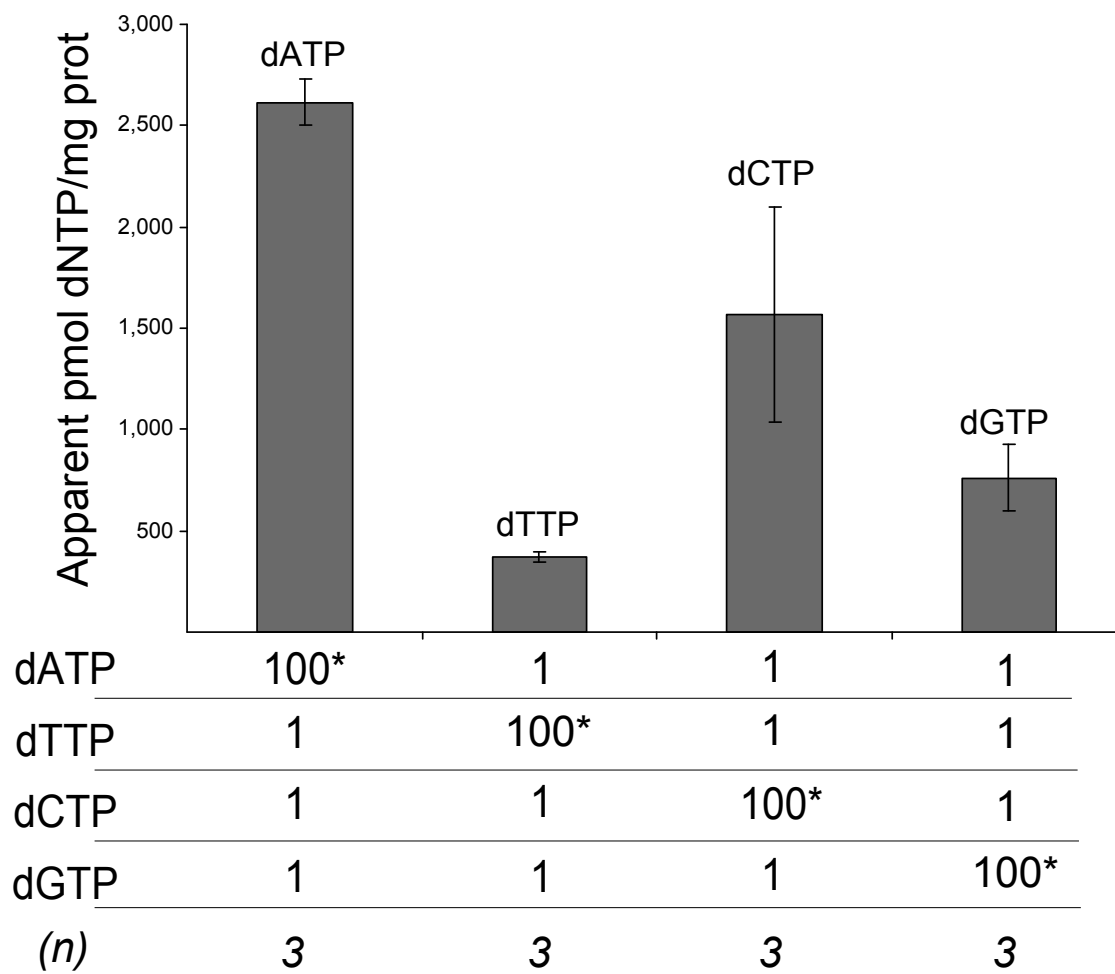

Supplement: Figure S1 — Transport of radioactive label from exogenous 100 µM [8-3H]dATP, [8-3H]dGTP, [5,5′-3H]dCTP or [methyl-3H]dTTP into mitochondria after 2 hours of in organello reaction. Radioactivity of the mitochondrial pellet was measured and apparent pmoles were estimated from the specific radioactivity of the dNTPs. Concentrations (µM) of dNTPs added to the reaction are indicated in the attached table. Asterisks: radiolabeled nucleotide. Bars represent mean±SD. (PDF) [file pgen.1002035.s001.pdf]

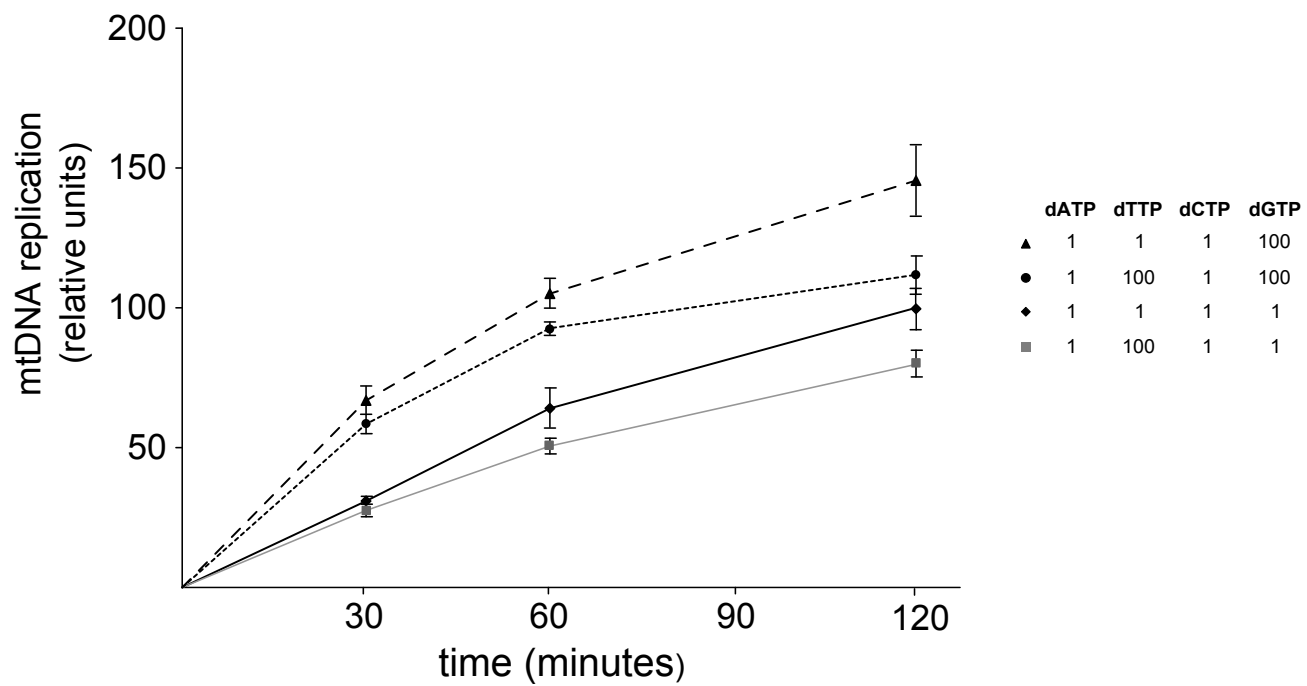

| Replication rate (relative units) |             |             |
|-----------------------------------|-------------|-------------|
|                                   | 0-30 min    | 60-120 min  |
| ▲                                 | 2.23 ± 0.17 | 0.67 ± 0.15 |
| ●                                 | 1.94 ± 0.10 | 0.32 ± 0.11 |
| ◆                                 | 1.03 ± 0.06 | 0.60 ± 0.12 |
| ■                                 | 0.92 ± 0.08 | 0.49 ± 0.04 |

Supplement: Figure S2 — Monitoring of mtDNA synthesis over 2 hours with dGTP and dTTP excess. Concentrations (µM) of dNTPs added to the reaction are indicated in the table on the right. The replication observed after 2 hours of reaction with 1 µM each dNTP added (rhombs) is considered the reference point (100%). Error bars represent ±SD (N = 3). Replication rates in the table at the bottom are expressed as the percentage of replication per minute. (PDF) [file pgen.1002035.s002.pdf]

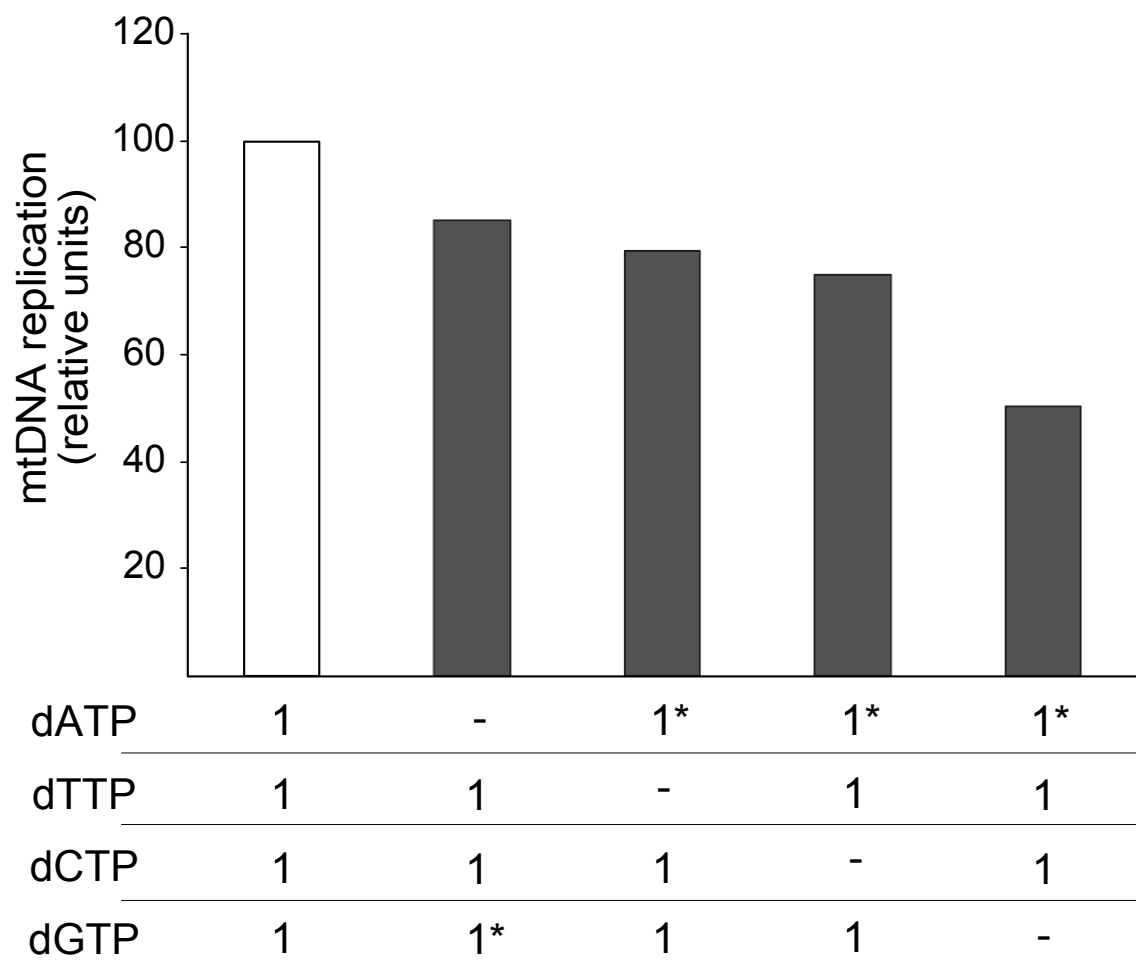

Supplement: Figure S3 — Effect of removing each single exogenous dNTP on mtDNA synthesis. Concentrations (µM) of dNTPs added to the reaction are indicated in the attached table. Dashes: no dNTP addition. Asterisks: radiolabeled nucleotide. Results were obtained after 2 hours of in organello reaction. (PDF) [file pgen.1002035.s003.pdf]

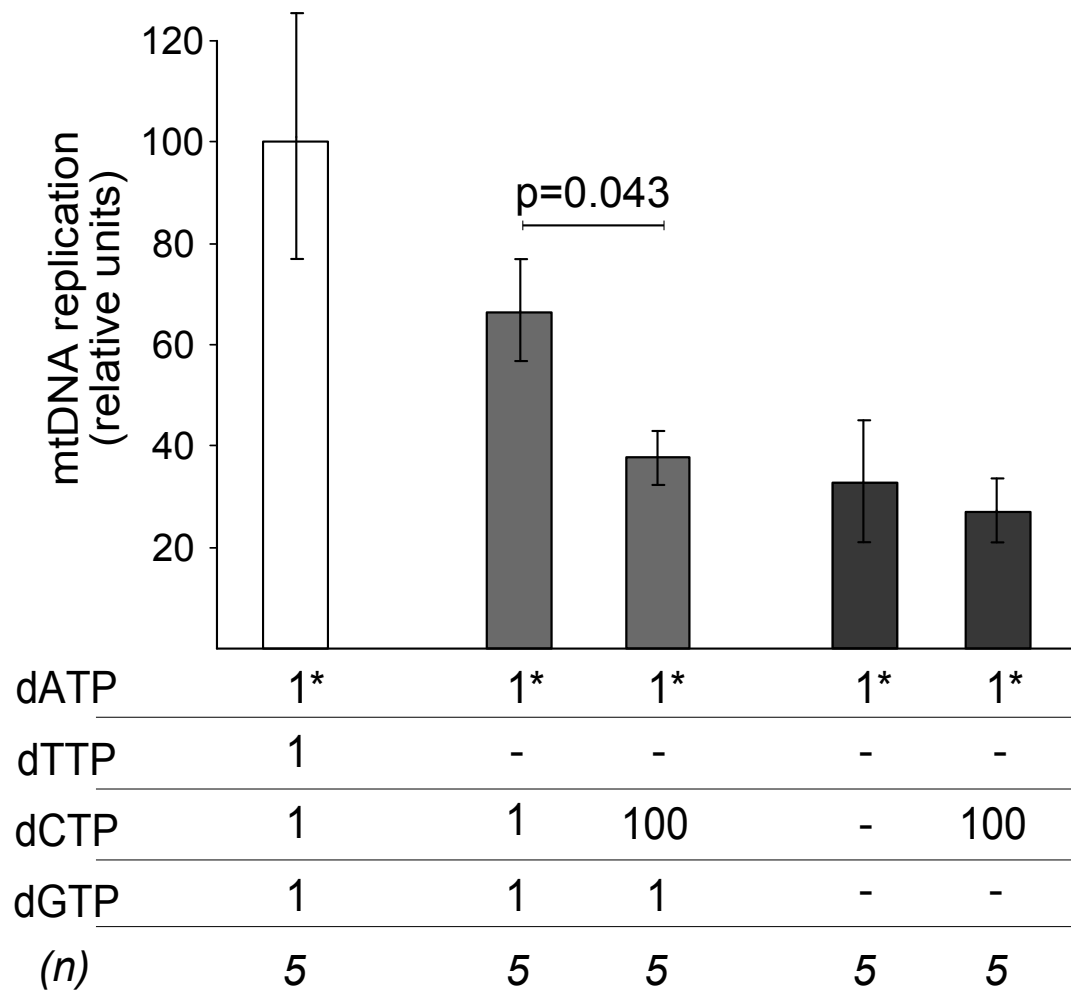

Supplement: Figure S4 — Effect of dCTP excess on mtDNA synthesis when exogenous dTTP (or dTTP and dGTP) was omitted in the in organello reaction. The dCTP-induced decrease of mtDNA synthesis disappeared when dGTP was omitted, likely because dGTP became the limiting substrate, as is suggested in Figure 2A and 2F, and Figures S2 and S3. Bars represent mean±SD. The reference result (open bar) is plotted as the mean of all the experiments, equaled to 100% and the error bar indicates the SD as percentage. P values obtained with the Wilcoxon T-test. (PDF) [file pgen.1002035.s004.pdf]
